# Using concept mapping in the knowledge-to-action process to compare stakeholder opinions on barriers to use of cancer screening among South Asians

**DOI:** 10.1186/1748-5908-8-37

**Published:** 2013-03-23

**Authors:** Rebecca Lobb, Andrew D Pinto, Aisha Lofters

**Affiliations:** 1 Centre for Research on Inner City Health, Keenan Research Centre, Li Ka Shing Knowledge Institute, St. Michael’s Hospital, 30 Bond St, Toronto, ON M5B 1W8, USA; 2 Division of Public Health Sciences, Department of Surgery and Alvin J. Siteman Cancer Center, Washington University School of Medicine, 660 S. Euclid, Campus Box 8100, St. Louis, MO 63110, USA; 3 Department of Family and Community Medicine, St. Michael’s Hospital, 30 Bond St, Toronto, ON M5B 1W8, USA

## Abstract

**Background:**

Using the knowledge-to-action (KTA) process, this study examined barriers to use of evidence-based interventions to improve early detection of cancer among South Asians from the perspective of multiple stakeholders.

**Methods:**

In 2011, we used concept mapping with South Asian residents, and representatives from health service and community service organizations in the region of Peel Ontario. As part of concept mapping procedures, brainstorming sessions were conducted with stakeholders (n = 53) to identify barriers to cancer screening among South Asians. Participants (n = 46) sorted barriers into groups, and rated barriers from lowest (1) to highest (6) in terms of importance for use of mammograms, Pap tests and fecal occult blood tests, and how feasible it would be to address them. Multi-dimensional scaling, cluster analysis, and descriptive statistics were used to analyze the data.

**Results:**

A total of 45 unique barriers to use of mammograms, Pap tests, and fecal occult blood tests among South Asians were classified into seven clusters using concept mapping procedures: patient’s beliefs, fears, lack of social support; health system; limited knowledge among residents; limited knowledge among physicians; health education programs; ethno-cultural discordance with the health system; and cost. Overall, the top three ranked clusters of barriers were ‘limited knowledge among residents,’ ‘ethno-cultural discordance,’ and ‘health education programs’ across surveys. Only residents ranked ‘cost’ second in importance for fecal occult blood testing, and stakeholders from health service organizations ranked ‘limited knowledge among physicians’ third for the feasibility survey. Stakeholders from health services organizations ranked ‘limited knowledge among physicians’ fourth for all other surveys, but this cluster consistently ranked lowest among residents.

**Conclusion:**

The limited reach of cancer control programs to racial and ethnic minority groups is a critical implementation issue that requires attention. Opinions of community service and health service organizations on why this deficit in implementation occurs are fundamental to understanding the solutions because these are the settings in which evidence-based interventions are implemented. Using concept mapping within a KTA process can facilitate the engagement of multiple stakeholders in the utilization of study results and in identifying next steps for action.

## Background

In Canada, cancer is the leading cause of mortality [1]. Ontario, the most populous province in Canada [2], has organized screening programs to promote the early detection of breast (est. 1990), cervical (est. 1997) and colorectal (est. 2007) cancers [3]. In order to maximize reach, these programs use evidence-based interventions (EBIs), including targeted invitations, facilitated appointment booking, reducing out-of-pocket costs [4,5], as well as public education and communication of test results to patients and providers [3]. Overall, self-reported recent use of mammograms (73%) and Pap tests (73%) in Ontario are similar to the country as a whole [6,7], and self-reported rates of fecal occult blood test (FOBT) use (50%) are higher in Ontario compared to other provinces [8]. However, the Ontario cancer screening programs have limited reach to immigrant populations compared to Canadian-born residents [9-15], which diminishes the effectiveness of these programs [16] and potentially leads to health inequities.

South Asians, including those from India, Pakistan, Afghanistan, Bangladesh, and Sri Lanka, are among the fastest growing immigrant groups in both Canada and Ontario [17]. Immigrants from South Asian countries are particularly vulnerable to being inadequately screened for all three types of cancer because they generally have incomes lower than the national average [18], and awareness about cancer screening in their countries of origin is typically poor [19-21]. Lofters *et al.* found that among immigrant groups cervical cancer screening rates were lower for South Asian immigrant women compared to Canadian-born women and immigrants who arrived before 1985, both for women aged 18 to 49 years (adjusted rate ratio (ARR) 0.81, 95% confidence interval (CI) 0.80, 0.82) and for women aged 50 to 66 years (ARR 0.67, 95% CI 0.65, 0.69) [22]. Another study found that a lower percentage of India-born Canadian residents compared to European-born Canadian residents had ever performed a breast self exam (58.6% versus 75.2%, respectively) [23]. Although South Asian specific colorectal cancer screening data are not available, screening rates for colorectal cancer are lower for all immigrant groups as compared to Canadian-born residents [24].

Studies that explored barriers to use of mammography, Pap tests, and FOBTs among racial and ethnic minority groups, including South Asians, have primarily focused on the perspectives of women who are eligible to receive these services and occasionally the perspective of the healthcare provider [23,25-34]. Relatively little is known about how stakeholders from various organization types view barriers to use of mammography, Pap tests, and FOBTs among racial and ethnic minority groups. Implementation frameworks suggest that successful implementation of EBIs is associated with organizational characteristics (*e.g.*, readiness to adopt, leadership, culture), and the context in which organizations exist (*e.g.*, legislation, continuity of funding, inter-organizational works) [35-40]. Therefore, by identifying organizational perspectives on barriers to cancer screening for racial and ethnic minority groups we can begin to understand how organizational factors might influence implementation of EBIs [41]. This knowledge can be used to design studies that will examine the effect of implementation strategies to improve organizational delivery of and resident participation in cancer screening programs for medically underserved populations. We report on research that is part of a multi-phase project with the overarching goal to reduce inequities in cancer screening for South Asian immigrants to Ontario by identifying effective strategies for increasing use of EBIs. In this manuscript, we describe concept mapping that was used to compare barriers to the use of mammograms, Pap tests, and FOBTs among South Asians from the perspective of stakeholders from organizations and South Asian residents, and discuss how results from concept mapping are being used within a knowledge-to-action (KTA) process to inform future phases of the project.

## Methods

### Implementation framework

The multiple phases of our project are guided by the KTA process that conceptualizes a relationship between knowledge creation and an action cycle to translate research into ‘real-world’ settings. KTA views knowledge creation as occurring through a funnel that includes the multitude of primary studies or information on a topic at the widest end, knowledge synthesis in the middle, and knowledge products (*e.g.*, EBIs) at the most narrow end. The action cycle is the process that leads to the implementation of EBIs. Based on numerous theories and frameworks of implementation, the action cycle consists of the following activities: identify a problem; identify the EBI relevant to the problem; adapt the identified EBI to the local context; assess barriers to using the EBI; select, tailor, and implement strategies to promote use of the EBI; monitor EBI use; evaluate the outcomes of using the EBI; and sustain ongoing use of EBI. The KTA process is dynamic and is accomplished through iterative exchanges between researchers and the end-users of research [37,42].

**Figure 1:**
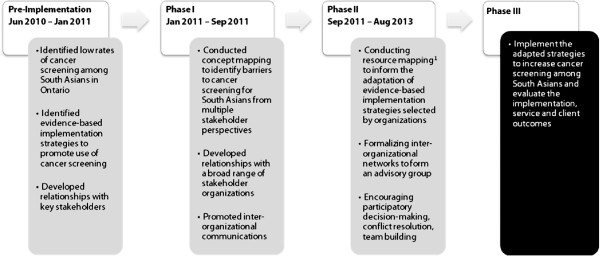
**Implementation framework for peel cancer screening study. **^1^Resource mapping includes use of geographic methods, semi-structured survey responses and an organizational network analysis. Gray boxes denote completed or on-going activities. Black boxes denote activities planned for the future.

Following an adapted KTA process, our project consists of a pre-implementation phase and three phases of implementation (Figure 1) [37]. In the pre-implementation phase, we identified low rates of cancer screening among South Asians in Ontario and implementation strategies that are effective to increase use of mammograms, Pap tests, and FOBTs. We developed relationships with three key stakeholders in our target setting including Cancer Care Ontario, the provincial authority for cancer screening programs in Ontario; the Medical Officer of Health in the region of Peel, an area of 1.2 million residents with cancer screening rates lower than other regions of Ontario [11], and a high concentration of immigrants from South Asia [43]; and the Executive Director of Punjabi Community Health Services, a community service organization that delivers culturally tailored health promotion services to South Asians in Mississauga and Brampton, the largest cities in the Peel region. These organizations were our initial community partners from the provincial, regional, and local levels respectively. Following funding from the Canadian Institutes of Health Research, we completed phase one (concept mapping) and launched phase two of the study. Knowledge gained from concept mapping is described in this paper. Future papers will describe findings from our current activities (phase two) and future activities (phase three).

### Study design

Concept mapping is a participatory research method that has been widely used for program planning [41,44-46]. This mixed methods approach uses qualitative procedures to generate data and quantitative methods to analyze data [41,44]. Concept mapping involves six steps: preparation, brainstorming, sorting and rating, analysis, interpretation, and utilization [47]. We conducted the preparation step in the pre-implementation phase of our project in collaboration with our initial community partners. During preparation we clarified the core issue to be addressed through concept mapping, developed the focus statements for brainstorming and rating sessions, and identified potential participants. The brainstorming, sorting, rating, analysis, and interpretation steps in concept mapping were conducted in phase one, and are described in this paper. The utilization step of concept mapping in which we are using concept mapping results to guide the selection, tailoring and implementation of interventions is being conducted in phase two of our study (Figure 1). Study activities have been approved by the St. Michael’s Hospital Research Ethic Board.

### Participants

To achieve a broad sampling of ideas about barriers to cancer screening for South Asians, we recruited 53 participants for brainstorming including potential decision makers, program implementers, and program participants from Brampton and Mississauga using a snowball sampling process that was initiated by our community collaborators. Punjabi Community Health Services recruited 24 South Asian immigrants to Canada through personal networks and existing health promotion programs. We recruited residents who spoke English, Punjabi or Urdu, the most common languages spoken among South Asians in Peel [43]. Translated invitation letters and consent forms were used for non-English speaking participants. South Asian resident participants ranged in age from 18 to 49 years (66%) to 50 to 69 years (34%), were male (36%) and female (64%), and spoke English (45%), Punjabi (38%) and Urdu (17%) as a primary language. Residents’ religious beliefs included Muslim (n = 4), Sikh (n = 14), Hindu (n = 5) and Christian (n = 1). Cancer Care Ontario’s Regional Primary Care Lead helped recruit 10 South Asian primary care physicians. A total of 13 organizations participated in brainstorming. Of the 13, seven were community service organizations (entities that routinely provide outreach and education to South Asian residents for the purpose of relocation assistance, health promotion, etc. but do not plan or provide cancer screening services), and six were health service organizations (public health, provincial health service program delivery, local clinical service delivery). Representatives from community service (seven senior managers, two health promoters, one case manager, one volunteer outreach coordinator) and health services organizations (four senior managers, two public health nurses, one project coordinator, one diversity support specialist) were identified through a collaborative effort by all partners.

From the group of participants in the brainstorming sessions, we invited residents that spoke English, primary care providers, and organizations to participate in sorting and rating. We also extended sorting and rating invitations to potential participants that were referred to us by participants in the brainstorming sessions. The 46 community members that participated in sorting and rating included South Asian residents (n = 15, eight men and seven women), five primary care providers and 17 organizations (11 community service, 6 health service). Representatives from community service organizations included: nine senior managers, two settlement counselors, one community services coordinator) and representatives from health service organizations included: eight senior managers, two health promoters, one community services coordinator, one project coordinator, one diversity support specialist, one public health nurse). They ranged in age from 18 to 49 years (70%) to 50 to 69 years (30%), and were female (70%) and male (30%). The lower number of participants in sorting and rating was due to lower participation by primary care providers and our decision to not recruit Punjabi and Urdu speaking residents because of budget constraints.

### Brainstorming

During brainstorming, participants worked in groups to generate statements in response to the focus prompt, ‘A barrier to use of mammograms, Pap tests, or fecal occult blood tests among South Asians in Peel is ______________?.’ We conducted ten brainstorming sessions. On the recommendation of our community partners, we held separate sessions for male and female residents, led by facilitators representing the same gender as participants, to minimize the discomfort of discussing personal health issues in a group. Brainstorming sessions in Urdu and Punjabi were led by lay facilitators from the community who had been trained by research staff. Following these sessions, the statements collected in Urdu and Punjabi were translated to English by qualified translators. The session with representatives from community organizations was held separately from the session with representatives from health service and public health organizations. The brainstorming sessions generated 290 statements. Two authors (RL and AP) used an independent review process with iterative meetings for comparison analyses to synthesize statements that were ascertained from multiple brainstorming sessions, reduce ideas to eliminate redundancy, and edit statements for clarity. The final list of statements included 45 unique barriers to use of mammograms, Pap tests, or FOBTs among South Asians in Peel.

### Sorting and rating

The majority of participants completed sorting and rating in-person with the exception of eight representatives from organizations who completed sorting and rating using the web-based Concept Systems software version 4.0175, Concept Systems, Inc. (Ithaca, NY). During sorting and rating sessions, the participants worked on an individual basis to sort statements into conceptually similar groups. Following the sorting activity, we administered four rating surveys to participants. Three surveys asked participants to rate each barrier based on, ‘How likely is it that addressing this barrier would increase the use of [specific test] among South Asians?,’ where the specific tests were mammograms, Pap tests, and FOBTs. A fourth survey measured the feasibility of addressing each barrier based on, ‘How strongly do you agree with the statement, It would be easy for the Peel community to remove this barrier within two to three years?.’ Response options were on a continuous scale for surveys one to three (1. extremely unlikely – 6. extremely likely) and survey four (1. strongly disagree – 6. strongly agree).

### Analysis

The analysis for sorting, rating, and comparison of ratings was also performed using the Concept Systems software. The software uses multi-dimensional scaling to create a point map based on sorting data. The point map shows the spatial relationships among statements with the goodness of fit indicated by a stress value. The stress value associated with our analysis was 0.2641, a value within acceptable limits for goodness of fit (<0.365) [16]. Details of the multi-dimensional scaling analysis are described in detail elsewhere [44,45]. Next, the software uses hierarchical cluster analysis to create a cluster map that partitions the statements on the point map into conceptual domains [44,45,47]. ‘No simple mathematical criterion is available by which a final number of clusters can be selected’ [45], p.13, because the ‘best’ number of clusters ‘depends on the level of specificity desired and the context at hand’ [48], p.316. Instead of using a statistical criterion to determine the final number of clusters, we asked experts in cancer control planning to examine different cluster solutions to interpret the best number of clusters and grouping of statements. This approach is standard for concept mapping [44,45,47]. It is important to note that the spatial position of the statements never change in the cluster map because the statement’s position is determined by the multi-dimensional scaling. However, the clusters, formed by circles around the statements, can be influenced by asking the concept system software for a specific solution (*e.g.*, ten-cluster, nine-cluster, etc.) or by using the software to place a specific statement in a specific cluster. The flexibility with deciding the final groupings for the statements invites the community to take ownership of the data and create information from the data that is meaningful to them [44,45,47]. Using the software, we created a visual display of the clusters (concept maps) with the statement numbers assigned by the concept mapping system. We computed the average ratings for each barrier and each cluster of barriers, and estimated the simple linear correlation in average ratings for groups using the Pearson correlation coefficient (r^2^). We report average ratings across subgroups of residents, and representatives from community service and health service organizations.

### Interpretation

**Figure 2:**
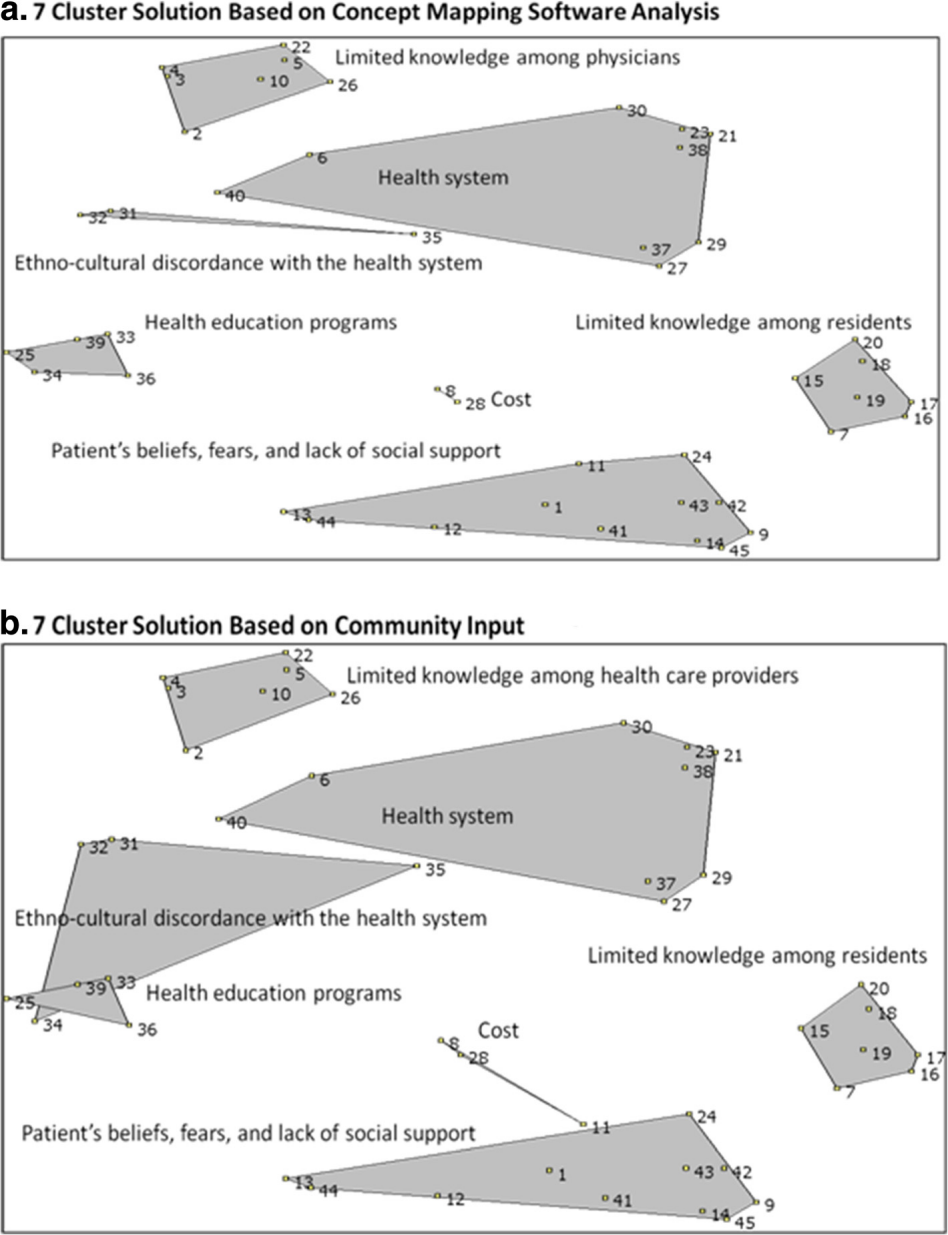
**Cluster maps. a.** 7 Cluster Solution Based on Concept Mapping Software Analysis. **b.** 7 Cluster Solution Based on Community Input.

We used a research-processing stage and participant-processing stage to interpret the final number of clusters and grouping of statements [47]. The research-processing stage consisted of two study investigators (RL and AP) examining different cluster solutions and observing the clusters that were merged from the upper limit of clusters (n = 10) to the lowest limit (n = 5) [44,48]. The study investigators decided to present a seven-cluster solution to stakeholders in the participant-processing stage because the statements within the clusters were conceptually similar to each other and discrete from statements in other clusters (Figure 2a) [44].

The participant-processing stage consisted of an interpretation session during which study investigators (RL and AP) presented the seven-cluster solution to nine community leaders (one primary care physician, four representatives from community service organizations, and four representatives from public health and health service organizations). Four residents who attended earlier sessions were also invited to the interpretation session but were not able to attend due to work or school related commitments. Consistent with other concept mapping studies, community leaders examined each statement and discussed whether the number of clusters and statements within the clusters were most appropriate for program planning [44,45,47]. The community leaders agreed with the seven-cluster solution and the names for the clusters. Although, they expressed the opinion that some barriers could be representative of more than one cluster and suggested that we move two barriers to other clusters. As a result of this suggestion, we moved the barrier ‘education programs do not offer materials that are well translated and culturally appropriate’ (statement 34) from the cluster labeled ‘health education programs’ to ‘ethno-cultural discordance with the health system,’ and the barrier ‘patient is concerned about the cost associated with tests’ (statement 11) from ‘patient’s beliefs, fears, lack of social support’ to the cluster labeled ‘costs.’ By moving these statements the final concept map (Figure 2b) had overlapping clusters. While concept mapping ideally strives for a clustering solution that does not have overlapping clusters [44], the researchers felt it was more important to have the final map represent the community leaders’ viewpoints since they were ultimately the ones who would use the data.

## Results

### Cluster descriptions

The final list of barriers, statement numbers, and cluster descriptions are provided in Table 1. The clusters included: patient’s beliefs, fears, and lack of social support (eleven statements); cost (three statements); limited knowledge among residents (seven statements); ethno-cultural discordance with the health system (four statements); limited knowledge among physicians (seven statements); health education programs (four statements); and the health system (nine statements). The concept map (Figure 2b) shows the relationships among these clusters. The close proximity of health system, ethno-cultural discordance, and health education programs clusters on the concept map shows how barriers within these cluster are more related to each other than to barriers in clusters that are further away (limited knowledge among physicians or residents; cost; patients beliefs, fears, lack of social support). The smaller size of the limited knowledge among physicians, limited knowledge among residents and health education clusters indicates that barriers in these clusters were more frequently sorted together than the barriers in larger clusters (health system; ethno-cultural discordance; patient beliefs, fears lack of social support). The overlap in two clusters reflects the community leaders’ opinions on the conceptual overlap among statements in the ethno-cultural discordance and health education clusters.

**Table 1:** Concept mapping: community planning to reduce inequities in cancer screening

	**Patient’s beliefs, fears, and lack of social support**
14	Fear of emotional or physical discomfort about tests (e.g. pain, invasiveness, embarrassment or reluctance to handle feces)
42	Fear of the side effects of treatment (e.g. Loss of hair, loss of weight, pain, etc.)
45	Fear of going to the test alone
43	Belief about lack of confidentiality
1	Fear of starting a discussion about cancer or cancer screening with their physician
41	Fear that cancer will be detected (i.e. Stigma, neglect by family)
9	Fear about going to hospital
13	Female patient is not able to access cancer screening unless her partner approves
12	Religious belief about modesty
24	Lack of family and friends experienced with cancer screening to endorse participation
44	Females and their health are worthless in some families
	**Cost**
11	Patient is concerned about cost associated with specialized tests
28	Patient has difficulty accessing transportation, including cost
8	Patient experiences loss of time and wages to see the primary care provider
	**Limited Knowledge among Residents**
15	Limited knowledge about cancer screening tests
17	Limited accurate knowledge about cancer and risk factors
19	Limited knowledge about how to access tests
16	Limited knowledge about the success of cancer treatment
18	Limited knowledge about the Canadian health care system
20	Limited knowledge about using the health system when not sick
7	Patient does not prioritize cancer screening
	**Ethno-cultural discordance**
35	Health system does not respect or accommodate the culture and traditional notions of health care among South Asians
31	Not enough primary care providers and technicians from South Asian cultures or who speak South Asian languages
34	Education programs do not offer materials that are well translated and culturally appropriate
32	Not enough female primary care providers
	**Limited knowledge among physicians**
22	Primary care provider does not emphasize the need for cancer screening
5	Primary care provider does not equally emphasize the need for mammograms, Pap tests, and fecal occult blood tests
2	Primary care provider perceives a lower risk of cancer among South Asians
4	Primary care provider is unaware of guidelines for cancer screening
3	Primary care provider is unaware of cancer screening programs
10	Primary care provider lacks regard for patients’ personal choice about whether cancer screening should be completed
26	Primary care provider does not have financial incentive to ensure cancer screening is completed
	**Education programs**
25	Do not provide messages through multiple mediums accessed by South Asians (e.g. Newspaper, television, etc.)
33	Do not offer materials that are easy to understand (e.g. Use pictures to convey message, low reading level)
36	Do not offer endorsements from credible sources (e.g. places of worship, schools, South Asian cancer survivors)
39	Education programs sometimes deliver inconsistent messages
	**Health system**
40	Not enough partnerships between public health departments and primary care providers to promote cancer screening
23	The health system does not have automated reminders to prompt primary care providers to talk with patients about cancer screening
21	The health system does not provide personal reminders from a credible authority (e.g. Ministry of health)
27	Patient needs to access tests by going through a physician
30	The region of Peel does not have enough test facilities in convenient locations
37	Patient has limited time to talk about cancer screening with the primary care provider
29	Patient experiences delays in getting an appointment (e.g. Long wait, inconvenient times)
38	The health system sometimes discontinues successful cancer screening programs
6	The region of Peel does not have enough primary care providers

### Cluster ratings

Overall, three clusters consistently ranked highest for all surveys (importance for each of mammography, Pap test, and FOBT and feasibility): ‘limited knowledge among residents,’ ‘ethno-cultural discordance,’ ‘health education programs.’ Clusters of barriers related to ‘cost’ and ‘patient’s beliefs, fears, lack of social support’ consistently ranked as lowest in importance. There were a few instances when subgroups of participants differed in opinions on the three clusters with the highest ratings. ‘Cost’ ranked second among residents for the FOBT survey and ‘limited knowledge among physicians’ ranked third among representatives from health service organizations for the feasibility survey. Residents ranked ‘limited knowledge among physicians’ lowest for all surveys but this cluster was ranked third for the feasibility survey and fourth for all other surveys among representatives from health service organizations (Table 2).

**Table 2:** Ratings on barriers to cancer screening among south asians

	**Overall**	**Residents**^**1**^	**Community service organizations**^**2**^	**Health service organizations**^**3**^
	**n = 46**	**n = 15**	**n = 12**	**n = 19**
**Ranking (mean rating) mammogram survey**^**4**^				
Ethno-cultural discordance	1 (4.87)	2 (4.95)	1 (5.11)	1 (4.66)
Limited knowledge among residents	2 (4.77)	1 (4.96)	2 (5.01)	3 (4.47)
Health education programs	3 (4.76)	3 (4.88)	3 (4.94)	2 (4.55)
Health system	4 (4.40)	5 (4.60)	4 (4.66)	5 (4.09)
Cost	5 (4.24)	4 (4.86)	5 (4.58)	7 (3.53)
Limited knowledge among physicians	5 (4.24)	7 (4.33)	6 (4.35)	4 (4.10)
Patients’ beliefs, fears, lack of support	6 (4.18)	6 (4.56)	7 (4.21)	6 (3.85)
**Ranking (mean rating) pap test survey**^**4**^				
Ethno-cultural discordance	1 (4.89)	2 (4.88)	1 (5.24)	1 (4.68)
Health education programs	2 (4.80)	1 (4.98)	3 (5.10)	2 (4.47)
Limited knowledge among residents	3 (4.74)	3 (4.88)	2 (5.11)	3 (4.40)
Health system	4 (4.36)	5 (4.59)	4 (4.66)	5 (3.99)
Limited knowledge among physicians	5 (4.25)	7 (4.30)	5 (4.46)	4 (4.06)
Patients’ beliefs, fears, lack of support	6 (4.19)	6 (4.55)	7 (4.27)	6 (3.86)
Cost	7 (4.09)	4 (4.72)	6 (4.44)	7 (3.37)
**Ranking (mean rating) fecal occult blood test survey**^**4**^				
Health education programs	1 (4.70)	3 (4.71)	2 (5.02)	1 (4.50)
Limited knowledge among residents	2 (4.69)	1 (4.78)	3 (4.95)	2 (4.46)
Ethno-cultural discordance	3 (4.64)	4 (4.65)	1 (5.05)	3 (4.37)
Health system	4 (4.31)	5 (4.43)	4 (4.62)	5 (4.02)
Limited knowledge among physicians	5 (4.27)	7 (4.22)	6 (4.42)	4 (4.22)
Cost	6 (4.14)	2 (4.72)	5 (4.58)	7 (3.40)
Patients’ beliefs, fears, lack of support	7 (3.90)	6 (4.28)	7 (4.16)	6 (3.43)
**Ranking (mean rating) feasibility survey**^**5**^				
Health education programs	1 (4.93)	2 (5.07)	1 (4.96)	1 (4.80)
Limited knowledge among residents	2 (4.71)	3 (4.95)	3 (4.67)	2 (4.56)
Ethno-cultural discordance	3 (4.49)	1 (5.10)	2 (4.81)	4 (3.82)
Limited knowledge among physicians	4 (4.36)	7 (4.48)	4 (4.36)	3 (4.28)
Health system	5 (4.25)	5 (4.73)	5 (4.33)	5 (3.81)
Cost	6 (4.10)	4 (4.92)	7 (3.89)	6 (3.58)
Patients’ beliefs, fears, lack of support	7 (3.87)	6 (4.72)	6 (3.96)	7 (3.14)

1. Residents include male and female immigrants from South Asian countries (e.g. India, Pakistan, Afghanistan, Bangladesh, and Sri Lanka).

2. Community service organizations include administrators and staff from business that routinely provide outreach and education to South Asian residents for the purpose of relocation assistance, health promotion, or other social services but do not provide cancer screening services.

3. Health service organizations include staff from the regional department of public, administrators from health service organizations and primary care providers.

4. Question: How likely is it that addressing this barrier would increase the use of [specific test] among South Asians? Scale 1–6: extremely unlikely, very unlikely, unlikely, likely, very likely, extremely likely.

5. Question: How strongly do you agree with the statement, It would be easy for the Peel community to remove this barrier within 2–3 years? Scale 1–6: strongly disagree, disagree, somewhat disagree, somewhat agree, agree, strongly agree.

We found the correlation in ratings for clusters among residents and representatives from health service organizations was substantially weaker compared to the correlation in ratings for clusters for other bivariate comparisons (Table 3). The weakest correlation in ratings for clusters was for residents and representatives from health service organizations for the feasibility survey (r^2^ = 0.24). Correlations in ratings for clusters among residents and representatives from community service organizations were strong for the cancer screening surveys (r^2^ = 0.80-0.84), and relatively weak for the feasibility survey (r^2^ = 0.54). The correlation in ratings for clusters among representatives from community service and health service organizations was r^2^ = 0.77 or higher for all surveys.

**Table 3:** **Correlations**^**1 **^**in average ratings for clusters of barriers by key stakeholders**

**Type**	**Residents**^**2**^	**Community service organizations**
**Mammogram survey**^**5**^		
Community Org^3^	0.84	
Health Service Org^4^	0.42	0.77
**Pap test survey**^**5**^		
Community Org	0.80	
Health Service Org	0.50	0.86
**Fecal occult blood test survey**^**5**^		
Community Org	0.80	
Health Service Org	0.31	0.77
**Feasibility survey**^**6**^		
Community Org	0.54	
Health Service Org	0.24	0.78

1. Pearson’s correlation coefficient.

2. Residents include male and female immigrants from South Asian countries (e.g. India, Pakistan, Afghanistan, Bangladesh, and Sri Lanka).

3. Community service organizations include administrators and staff from business that routinely provide outreach and education to South Asian residents for the purpose of relocation assistance, health promotion, or other social services but do not provide cancer screening services.

4. Health service organizations include staff from the regional department of public, administrators from health service organizations and primary care providers.

5. Question: How likely is it that addressing this barrier would increase the use of [specific test] among South Asians? Scale 1–6: extremely unlikely, very unlikely, unlikely, likely, very likely, extremely likely.

6. Question: How strongly do you agree with the statement, It would be easy for the Peel community to remove this barrier within 2–3 years? Scale 1–6: strongly disagree, disagree, somewhat disagree, somewhat agree, agree, strongly agree.

## Discussion

The limited reach of population-based cancer control programs to racial and ethnic minority groups is a critical implementation issue that requires attention. Many studies have explored barriers to cancer screening from the perspective of women and some have examined the perspectives of primary care providers. However, the perspectives of representatives from stakeholder organizations are equally as important given that successful implementation of EBIs is associated with the inner context of organizations (*e.g.*, readiness to adopt, leadership, culture), individuals within organizations (*e.g.*, values, social networks perceived need for change), and the outer context such as sociopolitical factors (*e.g.*, legislations, monitoring and review), funding (*e.g.*, grants and continuity of funding), client advocacy (*e.g.*, consumer organizations, lawsuits), and inter-organizational networks (*e.g.*, professional organizations, leadership ties, communication) [35-40]. To date, the research on strategies to improve use of cancer screening shows that greater reach among racial and ethnic groups can be achieved when programs take into account the language and cultural characteristics of the target population, provide support to reduce logistical barriers (*e.g.*, transportation, appointment making), and use multi-level strategies [4,5,34,49]. However, whether these strategies are adopted will depend on organizational perspectives on the related barriers.

Little is known about the perspectives of representatives from health service (*e.g.*, community health centers, primary care providers, hospitals, mammography facilities, public health) and community service organizations (*e.g.*, health and fitness groups, settlement agencies, *et al*.) on barriers to use of mammography, Pap tests, and FOBTs among racial and ethnic minority groups. This study contributes new knowledge to implementation research in this area by examining which barriers are viewed by representatives from stakeholder organizations as most important and feasible to address to increase cancer screening among South Asians. We found considerable agreement among residents and representatives from organizations on the importance of the top barriers to cancer screening for South Asians. Notwithstanding the concurrence of these opinions, overall agreement in the ranking of clusters of barriers by residents and representatives from health service organizations was low. In particular, rankings were discordant for barriers associated with ‘cost’ and ‘limited knowledge among physicians,’ which suggest that important factors could be overlooked if only one stakeholder opinion is taken into account when planning health promotion programs. For example, if program developers prioritize implementation strategies to remove barriers to cancer screening based only on the perspective of South Asian residents then the need to address ‘limited knowledge among physicians’ might be overlooked, despite physician recommendation being among the strongest predictors of cancer screening [13,50-54].

We found that residents’ perspectives on which barriers to cancer screening are most important to address and feasible to change are more closely aligned with representatives from community service organizations than health services organizations. This finding could be due to the similar ethnic characteristics of residents and employees of community service organizations. In addition, representatives from community service organizations gain a broader understanding of their clients’ perspectives through ongoing discussions about social and economic factors, even at the level of executive director because of the ‘hands-on’ nature of this role in small organizations. In contrast, communication between clients and representatives from health service organizations tends be limited to biomedical characteristics of clients, and client contact is limited to clinical and clinical support staff.

Furthermore, our results suggest opportunities for health service and community service organizations to work together to remove ethno-cultural barriers to cancer screening for South Asians. Representatives from both types of organizations ranked the ‘ethno-cultural discordance’ cluster among the top three important barriers for all three cancer screening tests. Yet only representatives from community service organizations ranked ethno-cultural discordance as feasible to address. We interpret this pattern of responses as being reflective of the expertise in community service organizations to address ethno-cultural barriers to cancer screening for South Asians and the lack of this expertise in health service organizations. Community service organizations are generally staffed by employees who are culturally representative of the clients they serve and have skills in interpretation and translation of medical information. In addition, foreign trained medical professionals often work in community service organizations because it is difficult for them to gain accreditation in the Canadian health system [55]. This is among the reasons why health service organizations are generally understaffed in employees who are culturally representative of the populations they serve. Recently, Canada implemented strategies to improve the timely assessment and recognition of foreign trained medical personnel including bridge-to-licensure programs for licensed practical nurses, medical radiation technologists, and physicians [56]. However, this gap in skills among employees of health organizations highlights opportunities for collaboration with community service organizations to remove ethno-cultural barriers to cancer screening for South Asians.

Findings from our study inform the field of implementation science by identifying ways in which stakeholders’ opinions about barriers to use of an EBI can differ. In addition, our analysis highlighted potential strategies by which these differences could be used to address barriers to cancer screening for South Asians. Because our study uses the KTA framework, our findings also contribute to the action cycle through which research is translated to action.

Through the participatory processes of concept mapping our community advisory group has grown from the initial three partners to 12 organizations. In phase two of this study we are engaging in multiple activities with the advisory group to utilize the concept mapping results. First, we discussed potential EBIs, based on the Guide to Community Preventive Services [4,5,57], to address the top three barriers to cancer screening that were identified by the community and barriers in the ‘limited knowledge among physicians’ cluster that were identified as important by health service organizations. From these discussions, we identified resources to support implementation of patient targeted, physician targeted, and health system targeted interventions. For example, a lay health advisor intervention is being developed with support from the Canadian Cancer Society. This intervention will include group education sessions for residents at local community service organizations and potentially South Asian screening clinics or blocked times for appointments with female physicians. Using logic models researchers and members of the advisory group developed a shared understanding of resources, activities, outputs, and outcomes for a multi-level intervention program (patient, provider, and health system level) to increase cancer screening among South Asians in Peel. To further inform the availability of and gaps in resources to support these interventions, we conducted a survey of all community service and health service organizations that provide services to promote cancer screening in Peel. When analyzed, the survey will inform us about the types of services organizations provide to promote cancer screening (*e.g.*, outreach and education, navigation, clinic services), the inter-organizational relationships (*e.g.*, communication, referral, collaboration) that support the delivery of the services, and the gaps in services that we need to fill through additional partnerships and resources. Following our accomplishment of a clearly defined intervention to improve rates of cancer screening among South Asians, we will seek funding for phase three in which we will examine the effect of change strategies on implementation of the multi-level cancer screening program.

Despite the strengths of this study, some limitations should be noted. We had limited participation from primary care physicians in the sorting and rating phase (n = 5) and no participation from residents in the interpretation phase. However, the impact of this limitation is minimal for two reasons. First, we were primarily interested in the organizational level perspective, not specifically primary care provider opinions, and physicians represented 26% (5/19) of the responses from representatives from health service organizations. Second, residents might not have felt comfortable speaking their opinions with a group of community leaders in the interpretation session. Fortunately, the multi-phase nature of our project will allow us to seek input on program development from South Asian residents at another point in the study. The generalizability of our findings to other provinces in Canada, to other countries, or healthcare settings may be limited because the perceptions of which barriers are most important and feasible to address will be influenced by local health policy, infrastructure, and practices. However, the methods used to conduct our study can be applied in other settings, and the general differences in opinion that we observed among stakeholders groups are likely representative of what we would find in other regions.

By using concept mapping, we identified barriers to cancer screening in the region of Peel that can be utilized in latter stages of the KTA process. Equally important was that concept mapping engaged a diverse range of stakeholders from the national level (*e.g.*, Canadian Cancer Society), provincial level (Cancer Care Ontario), regional level (Peel Public Health, regional cancer center) and local level (*e.g.*, hospitals, community health centers, community service organizations) that will make the implementation process relevant, feasible, and sustainable moving forward [42]. Participatory research methods combined with an overarching KTA framework can facilitate the translation of research to action.

## Competing interests

The authors declare that they have no competing interests to disclose. Funding for support of Drs. Lobb, Pinto, and Lofters work on this project was provided by the Canadian Institutes for Health Research, and the Ontario Ministry of Health and Long-Term Care.

## Authors’ contributions

RL contributed to the theoretical background, conceptualization of the study, supervised the acquisition, analyses, and interpretation of the data, had the final approval of the version of the manuscript to be published. AP contributed to the acquisition, analysis, and interpretation of the data, and provided important intellectual content to the preparation of the manuscript. AL contributed to the conceptualization of the study, analysis, and interpretation of the data, and provided important intellectual content to the preparation of the manuscript. All authors read and approved the final manuscript.

## References

[B1] Statistics Canada Leading causes of death CANSIM table 102–0561 and Catalogue no 84-215-X 2010 http://www.statcan.gc.ca/tables-tableaux/sum-som/l01/cst01/hlth36a-eng.htm Accessed March 27, 2013

[B2] Canadian Census Highlights Fact Sheet 7, Immigration and Citizenship, Ministry of Finance 2006 http://www.fin.gov.on.ca/en/economy/demographics/census/cenhi06-7.pdf *Accessed on March 27, 2013*

[B3] Cancer Care Ontario Types of screening programs http://www.cancercare.on.ca/pcs/screening/*Accessed March 27, 2013*

[B4] Baron RC Rimer BK Breslow RA Coates RJ Kerner J Melillo S Habarta N Kalra GP Chattopadhyay S Wilson KM Client-directed interventions to increase community demand for breast, cervical, and colorectal cancer screening a systematic review Am J Prev Med 2008 35 S34 55 10.1016/j.amepre.2008.04.002 18541187

[B5] Baron RC Rimer BK Coates RJ Kerner J Kalra GP Melillo S Habarta N Wilson KM Chattopadhyay S Leeks K Task Force on Community Preventive S Client-directed interventions to increase community access to breast, cervical, and colorectal cancer screening a systematic review Am J Prev Med 2008 35 S56 66 10.1016/j.amepre.2008.04.001 18541188

[B6] Shields M Wilkins K An update on mammography use in Canada Health Rep 2009 20 7 19 19813435

[B7] Chiarelli AM Halapy E Nadalin V Shumak R O’Malley F Mai V Performance measures from 10 years of breast screening in the Ontario Breast Screening Program, 1990/91 to 2000 Eur J Cancer Prev 2006 15 34 42 10.1097/01.cej.0000195713.02567.36 16374227

[B8] Wilkins K Shields M Colorectal cancer testing in Canada–2008 Health Rep 2009 20 21 30 19813436

[B9] Katz SJ Hofer TP Socioeconomic disparities in preventive care persist despite universal coverage Breast and cervical cancer screening in Ontario and the United States. Jama 1994 272 530 534 8046807

[B10] Lofters AK Glazier RH Agha MM Creatore MI Moineddin R Inadequacy of cervical cancer screening among urban recent immigrants: a population-based study of physician and laboratory claims in Toronto, Canada Prev Med 2007 44 536 542 10.1016/j.ypmed.2007.02.019 17467782

[B11] Krzyzanowska MK Barbera L Elit L Kwon J Lofters A Saskin R Yeritsyan N Bierman AS Bierman AS Cancer Project for an Ontario Women's Health Evidence-Based Report: Volume 1 2009 Toronto: St. Michael's Hospital and the Institute for Clinical Evaluative Sciences

[B12] Glazier RH Creatore MI Gozdyra P Matheson FI Steele LS Boyle E Moineddin R Geographic methods for understanding and responding to disparities in mammography use in Toronto, Canada J Gen Intern Med 2004 19 952 961 10.1111/j.1525-1497.2004.30270.x 15333060 PMC1492521

[B13] Hanson K Montgomery P Bakker D Conlon M Factors influencing mammography participation in Canada: an integrative review of the literature Curr Oncol 2009 16 65 75 10.3916/c32-2009-02-005 19862363 PMC2768512

[B14] McDonald JT Kennedy S Cervical cancer screening by immigrant and minority women in Canada J Immigr Minor Health 2007 9 323 334 10.1007/s10903-007-9046-x 17345152

[B15] Woltman KJ Newbold KB Immigrant women and cervical cancer screening uptake: a multilevel analysis Can J Public Health 2007 98 470 475 19039885 10.1007/BF03405441 PMC6975610

[B16] Kane M Trochim WMK Concept Mapping for Planning and Evaluation 2007 Inc.: Sage Publications

[B17] Statistics Canada Visible minority population by province and territory (2006 Census of Population) http://www.statcan.gc.ca/tables-tableaux/sum-som/l01/cst01/demo52b-eng.htm Accessed March 27, 2013

[B18] Statistics Canada The South Asian Community in Canada 2001 http://www.statcan.gc.ca/pub/89-621-x/89-621-x2007006-eng.htm Accessed March 27, 2013

[B19] Banning M Hafeez H Perceptions of breast health practices in Pakistani Muslim women Asian Pac J Cancer Prev 2009 10 841 847 20104976

[B20] Imam SZ Rehman F Zeeshan MM Maqsood B Asrar S Fatima N Aslam F Khawaja MR Perceptions and practices of a pakistani population regarding cervical cancer screening Asian Pac J Cancer Prev 2008 9 42 44 18439071

[B21] Kumar Y Mishra G Gupta S Shastri S Cancer screening for women living in urban slums - acceptance and satisfaction Asian Pac J Cancer Prev 2011 12 1681 1685 22126544

[B22] Lofters AK Hwang SW Moineddin R Glazier RH Cervical cancer screening among urban immigrants by region of origin: a population-based cohort study Prev Med 2010 51 509 516 10.1016/j.ypmed.2010.09.014 20932995

[B23] Brotto LA Chou AY Singh T Woo JS Reproductive health practices among Indian, Indo-Canadian, Canadian East Asian, and Euro-Canadian women: the role of acculturation J Obstet Gynaecol Can 2008 30 229 238 18364100 10.1016/S1701-2163(16)32759-1

[B24] Sewitch MJ Fournier C Ciampi A Dyachenko A Colorectal cancer screening in Canada: results of a national survey Chronic Dis Can 2008 29 9 21 19036219

[B25] Ahmad F Mahmood S Pietkiewicz I McDonald L Ginsburg O Concept mapping with South Asian immigrant women: barriers to mammography and solutions Journal of immigrant and minority health / Center for Minority Public Health 2012 14 242 250 10.1007/s10903-011-9472-7 21538023

[B26] Basu P Sarkar S Mukherjee S Ghoshal M Mittal S Biswas S Mandal R Sankaranarayanan R Women’s perceptions and social barriers determine compliance to cervical screening: results from a population based study in India Cancer Detect Prev 2006 30 369 374 10.1016/j.cdp.2006.07.004 16963194

[B27] Ahmad F Cameron JI Stewart DE A tailored intervention to promote breast cancer screening among South Asian immigrant women Soc Sci Med 2005 60 575 586 10.1016/j.socscimed.2004.05.018 15550305

[B28] Bottorff JL Johnson JL Bhagat R Grewal S Balneaves LG Clarke H Hilton BA Beliefs related to breast health practices: the perceptions of South Asian women living in Canada Soc Sci Med 1998 47 2075 2085 10.1016/S0277-9536(98)00346-3 10075248

[B29] Boxwala FI Bridgemohan A Griffith DM Soliman AS Factors associated with breast cancer screening in Asian Indian women in metro-Detroit J Immigr Minor Health 2010 12 534 543 10.1007/s10903-009-9277-0 19629691 PMC4276127

[B30] Choudhry UK Srivastava R Fitch MI Breast cancer detection practices of south Asian women: knowledge, attitudes, and beliefs Oncol Nurs Forum 1998 25 1693 1701 9826837

[B31] Donnelly TT Challenges in providing breast and cervical cancer screening services to Vietnamese Canadian women: the healthcare providers’ perspective Nurs Inq 2008 15 158 168 10.1111/j.1440-1800.2008.00409.x 18476858

[B32] Gurm BK Stephen J MacKenzie G Doll R Barroetavena MC Cadell S Understanding Canadian Punjabi-speaking South Asian women’s experience of breast cancer: a qualitative study Int J Nurs Stud 2008 45 266 276 10.1016/j.ijnurstu.2006.08.023 17049350

[B33] Ahmad F Jandu B Albagli A Angus JE Ginsburg O Exploring ways to overcome barriers to mammography uptake and retention among South Asian immigrant women Health & social care in the community 2013 21 88 97 10.1111/j.1365-2524.2012.01090.x 23057604

[B34] Hou SI Sealy DA Kabiru CW Closing the disparity gap: cancer screening interventions among Asians–a systematic literature review Asian Pacific journal of cancer prevention: APJCP 2011 12 3133 3139 22394003

[B35] Aarons GA Hurlburt M Horwitz SM Advancing a conceptual model of evidence-based practice implementation in public service sectors Adm Policy Ment Health 2011 38 4 23 10.1007/s10488-010-0327-7 21197565 PMC3025110

[B36] Kitson AL Rycroft-Malone J Harvey G McCormack B Seers K Titchen A Evaluating the successful implementation of evidence into practice using the PARiHS framework: theoretical and practical challenges Implementation science: IS 2008 3 1 10.1186/1748-5908-3-1 18179688 PMC2235887

[B37] Graham ID Logan J Harrison MB Straus SE Tetroe J Caswell W Robinson N Lost in knowledge translation: time for a map? J Contin Educ Health Prof 2006 26 13 24 10.1002/chp.47 16557505

[B38] Glisson C Schoenwald SK The ARC organizational and community intervention strategy for implementing evidence-based children’s mental health treatments Ment Health Serv Res 2005 7 243 259 10.1007/s11020-005-7456-1 16320107

[B39] Lobb R Colditz GA Implementation science and its application to population health Annual review of public health 2013 34 235 251 10.1146/annurev-publhealth-031912-114444 23297655 PMC3901430

[B40] Greenhalgh T Robert G Macfarlane F Bate P Kyriakidou O Diffusion of innovations in service organizations: systematic review and recommendations Milbank Q 2004 82 581 629 10.1111/j.0887-378X.2004.00325.x 15595944 PMC2690184

[B41] Green AE Aarons GA A comparison of policy and direct practice stakeholder perceptions of factors affecting evidence-based practice implementation using concept mapping Implementation science: IS 2011 6 104 10.1186/1748-5908-6-104 21899754 PMC3178500

[B42] Lobb R Petermann L Manafo E Keen D Kerner J Networking and Knowledge Exchange to Promote the Formation of Transdisciplinary Coalitions and Levels of Agreement Among Transdisciplinary Peer Reviewers J Public Health Manag Pract 2012 10.1097/PHH.0b013e31823991c2 22990496

[B43] Mowat D Stratton J A Picture of Health A Comprehensive Report on Health in Peel Chapter 4: Who Lives in Peel Region? 2008 http://www.peelregion.ca/health/health-status-report/chsr/chapter8.htm *Accessed on December 3, 2011* 2008

[B44] Trochim WM An Introduction to Concept Mapping for Planning and Evaluation Eval Program Plann 1989 12 1 16 10.1016/0149-7189(89)90016-5

[B45] Trochim WM Milstein B Wood BJ Jackson S Pressler V Setting objectives for community and systems change: an application of concept mapping for planning a statewide health improvement initiative Health Promot Pract 2004 5 8 19 discussion 10 14965431 10.1177/1524839903258020

[B46] O’Campo P Burke J Peak GL McDonnell KA Gielen AC Uncovering neighbourhood influences on intimate partner violence using concept mapping J Epidemiol Community Health 2005 59 603 608 10.1136/jech.2004.027227 15965146 PMC1757065

[B47] Burke JG O’Campo P Peak GL Gielen AC McDonnell KA Trochim WM An introduction to concept mapping as a participatory public health research method Qual Health Res 2005 15 1392 1410 10.1177/1049732305278876 16263919

[B48] Jackson K Trochim WMK Concept Mapping as an Alternative Approach for the Analysis of Open-Ended Survey Responses Organ Res Methods 2002 5 307 336 10.1177/109442802237114

[B49] Lu M Moritz S Lorenzetti D Sykes L Straus S Quan H A systematic review of interventions to increase breast and cervical cancer screening uptake among Asian women BMC Publ Health 2012 12 413 10.1186/1471-2458-12-413 PMC3488494 22676147

[B50] Cronan TA Villalta I Gottfried E Vaden Y Ribas M Conway TL Predictors of mammography screening among ethnically diverse low-income women J Womens Health (Larchmt) 2008 17 527 537 10.1089/jwh.2007.0331 18447760

[B51] Lubetkin EI Santana A Tso A Jia H Predictors of cancer screening among low-income primary care patients J Health Care Poor Underserved 2008 19 135 148 10.1353/hpu.2008.0001 18263990

[B52] Richards CL Viadro CI Earp JA Bringing down the barriers to mammography: a review of current research and interventions Breast Dis 1998 10 33 44 15687562 10.3233/bd-1998-103-406

[B53] Wang JH Mandelblatt JS Liang W Yi B Ma IJ Schwartz MD Knowledge, cultural, and attitudinal barriers to mammography screening among nonadherent immigrant Chinese women: ever versus never screened status Cancer 2009 115 4828 4838 10.1002/cncr.24517 19645031 PMC2761518

[B54] Brouse CH Wolf RL Basch CE Facilitating factors for colorectal cancer screening Journal of cancer education: the official journal of the American Association for Cancer Education 2008 23 26 31 10.1080/08858190701818283 18444043

[B55] Kondro W Credentialing body needed for foreign-trained doctors CMAJ 2004 171 435 15337713 10.1503/cmaj.1041243 PMC514627

[B56] A Pan-Canadian Framework for the Assessment and Recognition of Foreign Qualifications. Forum of Labour Market Ministers. Cat. No.: HS4-91/2009E-PDF 2009 Her Majesty the Queen in Right of Canada 978--1-100-13857-2

[B57] Baron RC Melillo S Rimer BK Coates RJ Kerner J Habarta N Chattopadhyay S Sabatino SA Elder R Leeks KJ Intervention to increase recommendation and delivery of screening for breast, cervical, and colorectal cancers by healthcare providers a systematic review of provider reminders Am J Prev Med 2010 38 110 117 10.1016/j.amepre.2009.09.031 20117566

